# LABOR FORCE TRANSITIONS, INCOME CHANGES, AND POVERTY ENTRIES AMONG OLDER WORKERS: 2018–2023

**DOI:** 10.1093/geroni/igae098.2507

**Published:** 2024-12-31

**Authors:** Callie Freitag, Heather Hill

**Affiliations:** University of Wisconsin-Madison, Wisconsin, United States; University of Washington, Seattle, Washington, United States

## Abstract

Economic downturns and recoveries, like the one caused by the COVID-19 pandemic, can change retirement plans by prompting early retirement or inducing workers to remain in the labor force longer. These changes in the timing and circumstances of later-life labor force transitions can have significant impacts on the income sources available to those making the transition—and thus the likelihood of experiencing poverty—but prior studies do not examine these outcomes. Using the Current Population Survey March ASEC from 2018-2023, this study examines older adults’ (age 50 or over) labor force transitions during the COVID-19 pandemic. We contribute to prior literature by focusing specifically on trends, and the economic consequences of two types of labor force exits: retirement or non-retirement. Our main analysis estimates associations between labor force transitions and entries into poverty and describes changes in income during labor force transition years. We find that transitioning out of the labor force for any reason is significantly associated with substantial reductions in total income and a higher likelihood of entering poverty. However, these associations did not change much throughout the pandemic or recovery period. We also find that COVID-19 Economic Impact Payments and Unemployment Insurance were important protections against earnings losses and poverty entries in 2021, but especially for those who transitioned out of the labor force. This study also sheds light on the fluidity of retirement and labor force participation in later life.

